# A Distributed IoT Air Quality Measurement System for High-Risk Workplace Safety Enhancement

**DOI:** 10.3390/s23115060

**Published:** 2023-05-25

**Authors:** Lorenzo Parri, Marco Tani, David Baldo, Stefano Parrino, Elia Landi, Marco Mugnaini, Ada Fort

**Affiliations:** Department of Information Engineering and Mathematics, University of Siena, 53100 Siena, Italy

**Keywords:** gas sensors, sensor network, distributed measurement system, workplace safety, air quality

## Abstract

The safety of an operator working in a hazardous environment is a recurring topic in the technical literature of recent years, especially for high-risk environments such as oil and gas plants, refineries, gas depots, or chemical industries. One of the highest risk factors is constituted by the presence of gaseous substances such as toxic compounds such as carbon monoxide and nitric oxides, particulate matter or indoors, in closed spaces, low oxygen concentration atmospheres, and high concentrations of CO_2_ that can represent a risk for human health. In this context, there exist many monitoring systems for lots of specific applications where gas detection is required. In this paper, the authors present a distributed sensing system based on commercial sensors aimed at monitoring the presence of toxic compounds generated by a melting furnace with the aim of reliably detecting the insurgence of dangerous conditions for workers. The system is composed of two different sensor nodes and a gas analyzer, and it exploits commercial low-cost commercially available sensors.

## 1. Introduction

A distributed measurement system based on chemical sensors can play a crucial role in enhancing workplace safety by detecting the presence of harmful chemicals and alerting workers in real time. The system can consist of a network of sensor nodes strategically placed at different locations in the workplace, wirelessly connected to a central monitoring unit.

The sensor nodes can be designed to detect specific chemical compounds that pose a threat to worker safety, such as particulate matter, carbon monoxide, and nitric oxides, among others. The monitoring system can process the data from the sensors and provide real-time information about the concentration of harmful chemicals in the workplace, enabling workers to take appropriate actions to avoid exposure.

In addition, the system can provide long-term data analysis and trending, which can be used to identify areas where the workplace may need to be improved to better protect workers from chemical hazards. This information can also be used to support continuous improvement efforts, such as the optimization of ventilation systems or to detect plant malfunctions.

In this scenario, there are several challenging aspects concerning the design of a distributed measurement system based on chemical sensors.

The selection of the right sensors can be a challenging aspect because there are many different types of gas sensors available, each with its own unique set of capabilities and limitations [1]. The sensors must be able to accurately detect the presence of the targeted harmful chemicals in the workplace and provide reliable data to the monitoring unit.

Another fundamental aspect when working with chemical sensors is the accuracy maintained over time. Gas sensors can be affected by a variety of factors, including temperature, humidity, drift, and the presence of other chemical interferences, which can impact their accuracy [2,3]. Pre-calibrated gas sensors are rarely available on the market. Usually, sensors need to be calibrated regularly to ensure that they are providing accurate readings. The need for periodic calibrations can be a challenging aspect in a distributed system where the sensors are located in different parts of the workplace.

The selection of the best detection technology, among those available on the market, must take into account a series of characteristics such as those shown in Table 1.

In the last years, sensors are more accurate and reliable than ever before, and they can detect a wider range of harmful chemicals in the workplace [4]. Among available sensor technologies, electrochemical sensors [5] are the ones with a larger availability for many different gas species such as carbon monoxide CO, nitrogen dioxide NO_2_, oxygen, and many other chemical compounds. The main limitations of these sensors are the lifetime (usually no more than two years) and the possible cross-interference with other gases. Other low-cost and robust gas sensors are the semiconductor bases chemoresistive sensors [6,7]. These sensors usually have a very high sensitivity and a good lifetime but unfortunately, they usually have poor selectivity in the detection of different gas species. Selectivity in gas measurements is intended for the ability to detect a particular gas species without interferences caused by other species present in the mixture.

The detection of inert gases, such as carbon dioxide CO_2_, requires different sensing techniques such as optical methods. The most employed sensors are based on non-dispersive infrared technology (NDIR) [8]. These sensors are based on the adsorption of infrared radiation by the target gas and are characterized by a long lifetime and good selectivity but high energy demand due to the presence of an infrared source.

Regarding the state of the art, the development of distributed measurement systems based on chemical sensors for workplace safety enhancement has advanced significantly in recent years. Many factors have contributed to the development of ever more efficient and cheaper systems. First, the availability on the market of new and improved sensors has been a key factor in advancing the state of the art in this field.

From the architectural point of view, there exist many different solutions proposed in the literature for gas monitoring systems. In [9], a system measuring temperature, humidity, and volatile organic compounds (VOCs) with LoRa wireless data transmission is presented, in [10] a review of different wireless systems for air pollutant measurement is presented, in [11] a LoRa sensor network was developed for gas leakage event detection is presented, while in [12] an air quality measurement sensor network was specifically designed for urban environments. In the last years, the tendency is to develop wireless and cloud-based solutions like the ones presented in [13,14] and, when it is possible, battery-powered systems to facilitate installation on pre-existing plants like the one presented in [15]. The widespread adoption of wireless technologies has made it possible to create networks of sensors that can transmit data to a central monitoring unit in real time. These technologies have made it easier to implement distributed measurement systems that cover large areas and provide real-time data on chemical hazards. The growth of cloud computing has made it possible to store and process large amounts of data from a distributed measurement system in a central location. This enables real-time analysis of the data, and it allows access to information from anywhere with an internet connection [16]. Talking about commercial devices, the most common commercial solutions available on the market are designed for hazardous areas where there is a relevant risk of explosion due to the presence of flammable compounds as defined for example by the European ATEX directive. Examples of these products are the ones produced by *MSA* (Cranberry Township, PA, USA) or *Siemens* (Munich, Germany). There also exist portable battery-powered devices such as the *BW series by Honeywell* (Charlotte, North Carolina, USA) or *Testo 350 by Testo* (Titisee-Neustadt, Germany), whose battery life is usually limited to some hours of continuous operation. In terms of available devices, even if they are characterized by a relatively high cost, the market offers many opportunities for many different producers. However, in terms of data communications and system integrability, the design of a flexible and expandible platform using commercial devices is a hard task. In fact, a system that offers, for example, wireless communication, uses general proprietary protocols that may also require licensed software or special interfaces.

Exhaust gas analyzers are instruments that are available on the market for many years, and they are employed mostly for the measurement of combustion processes exhaust gases. The most accurate devices are the ones based on optical measurement principles such as NDIR [17] or ultraviolet (UV) detectors. An example is portable gas analyzers produced by *Horiba* (Kyoto, Japan) such as the *PG350* series. These instruments are extremely accurate and selective. The main limitation of this type of device is the calibration loss over time. An accurate gas analyzer requires frequent calibrations to maintain the declared accuracy. Systems designed to continuously monitor exhaust gas compositions are called continuous emission monitoring systems (CEMS) and are specifically designed to maintain calibration for a long time or to performs automatic periodic calibrations. Obviously, this last class of analyzers is very expensive and requires continuous and high-cost maintenances.

In this work, the authors propose a system composed of three fully custom-designed gas sensing systems for a particular application. The target application consists in a plant where a melting furnace is installed inside a building. The melting furnace is used to separate different metals exploiting their different melting temperature. The furnace is developed by *Italimpiant Orafi s.p.a* (Italy) a company based in Italy that produces lines for the recovery of precious and non-precious metals. The furnace is currently used for recovering gold and other metals from electronic boards or other scraps and in the near future, it will be used to recycle exhaust lithium battery cells.

In this application, the sensors need to continuously measure the concentration of carbon monoxide (CO), nitrogen dioxide (NO_2_), oxygen (O_2_), carbon dioxide (CO_2_), and particulate matter (PM). Sensors are hosted by two environmental sensor nodes (one installed inside the plant and one outside) and in an exhaust gas analyzer monitoring the exhaust gas produced by the melting furnace.

Considering the project requirements, electrochemical sensors were used for the detection of toxic compounds and oxygen. The choice of NDIR sensors for detecting carbon dioxide and laser scattering sensors for particulate matter was forced by the fact that they are the only ones available on the market for these composes.

The choice of electrochemical sensors for the devices developed in this work is not the optimal solution in terms of measurement accuracy, cross interferences, and sensor lifetime (usually not more than 2 years), but it results well in terms of calibration retention and sensor cost. However, electrochemical sensors that are commonly used for environmental monitoring, are not widely used for exhaust gas measurement for different reasons. The main issue is sensor poisoning in case of very high concentration exposure. Electrochemical sensors, if exposed to high concentrations, even for a short period of time, may require a long time to recover and this makes them not used to measure lower concentrations later, for long periods (minutes or hours). In this work, an ad hoc solution has been implemented to cope with this issue and allow for the use of electrochemical sensors in an exhaust gas analyzer.

The proposed system aims to merge the flexibility of a distributed wireless sensor network with an exhaust gas analyzer exploiting the same low-cost sensing technologies and allows for easy implementation of a complete and scalable monitoring system for industrial applications.

## 2. System Architecture

The proposed monitoring system is based on a set of three different devices. The aim is the monitoring of air quality inside and outside the plant where the furnace is operating and, at the same time, measuring the composition of exhaust gases produced by the furnace.

**Figure 1:**
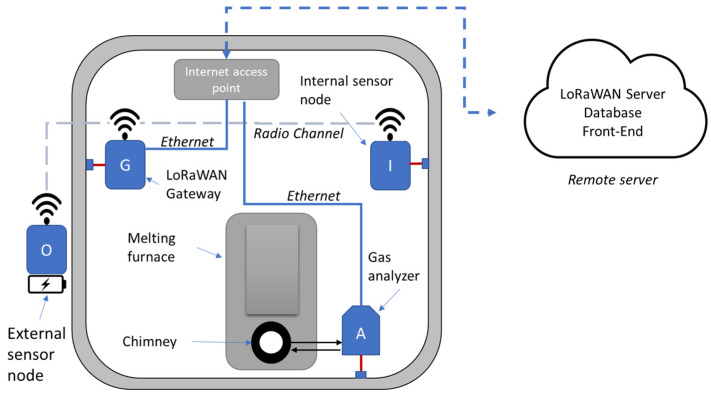
A scheme of the developed system composed of internal nodes I, the gas analyzer A, and the LoRaWAN gateway (inside the building where the furnace is hosted). Outside the building, there are the external nodes, O, that differently from the other system is battery powered.

As shown in Figure 1, the monitoring system is composed of two types of air quality measurement nodes, one type is conceived for monitoring the air quality inside the building able to measure the concentration of CO, NO_2_, oxygen, and CO_2_. This node is powered by the mains available in the building, the node transmits the measured concentrations through a LoRa wireless channel and a LoRaWAN protocol.

The second type of node is designed to operate outdoors, is battery operated, monitors the concentration of CO, NO_2_, and particulate matter (PM) and communicates through the same LoRa wireless channel as the indoor nodes.

Both nodes exploit the flexible architecture, based on the microcontroller unit (MCU) STM32L4Q5 by STMicroelectronics, described in [18], in which the gas sensors and their front-end electronics are hosted in a dedicated board called “sensor board”, connected to a second printed board called the “main board”, hosting the microcontroller, I/O device, the battery management unit, and the transceivers. This modular solution allows the adaptation of the node to different sensors, simply by re-designing the sensor board.

Regarding the data transmission strategy, as previously introduced, the sensor nodes exploit a LoRaWAN radio communication protocol, the nodes are standard class A LoRaWAN devices. Class A communication is always initiated by the end device so, in this case, the sensor node. The choice of LoRa modulation and LoRaWAN protocol was motivated by the need for a robust and long-range transmission system, also for indoor applications, with reduced power consumption. The drawback of using this protocol is the low bandwidth available. However, this limitation is not relevant to the scope of the project as the information to be transmitted is limited. A LoraWAN class A device can send an uplink message at any time respecting channel occupancy roles according to the standard. The two nodes, due to the different energy availability, transmit data messages at different intervals (1 min for the internal node and 60 min for the external node). Data from the sensor nodes are transmitted to a standard LoRaWAN gateway installed inside the building and connected to the internet network. The gateway communicates by the internet connection to a remote LoRaWAN server that manages the data and sends it to a database. The third device is a fully custom-designed gas analyzer that is used to measure the concentration of CO, NO, NO_2_, and O_2_, in the exhaust of the furnace, this device is powered by the main and it is directly connected to the internet by an ethernet connection.

The basic functionality of the whole monitoring system is to detect simultaneously the levels of the monitored gases inside and outside the building, and the composition of the exhaust gases while the furnace is working.

Thanks to the full custom development of node hardware, firmware, and remote server software, easy integration is possible with other existing systems, such as ventilation systems and fire suppression systems, to provide a comprehensive solution for workplace safety.

Regarding the sensors used, Table 2 shows the set of commercial devices used in sensor nodes and in the gas analyzer.

### 2.1. Internal Sensor Node

The internal and external sensor nodes have approximately the same structure. They are based on a microcontroller that acquires environmental data through a specific ad hoc designed electronic front end for each type of sensor (electrochemical, NDIR [19], particulate digital sensor).

The microcontroller exploits a LoRa radio module to transmit data to the gateway. The main difference between internal and external nodes is in the power supply system. The internal node is powered by a 5 V DC power supply directly connected to the mains. Regarding the sensor’s front end, for electrochemical sensors custom electronics for interfacing amperometric sensors are used while for infrared sensors the front end embeds a driver for the infrared (IR) source and an electronic front end for pyroelectric detectors [19].

An important difference between internal and external sensor nodes is the power management and data acquisition strategy. The internal node that has the availability of a 5 V power supply is continuously operating and it transmits data within a period of 60 s, whereas the external node switches between sleep mode and operations, and its data transmission rate is much lower in the order of transmission/hour to grant a sufficient battery duration, as will be discussed in detail in the following.

**Figure 2:**
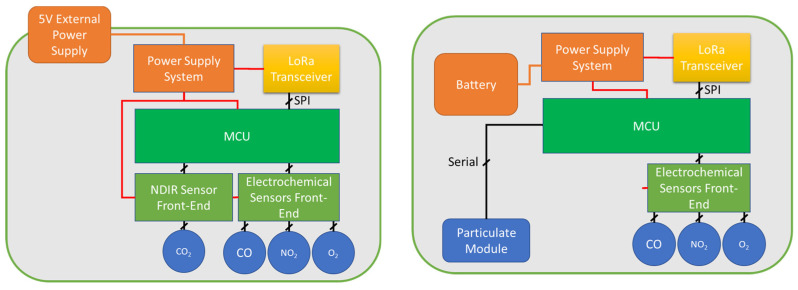
A block diagram of the two sensor nodes, the internal sensor (**left**) does not use battery power and particulate sensor module while the external sensor (**right**) does not use NDIR sensor and is powered by battery source.

A block diagram describing the architecture of both sensor nodes is shown in Figure 2.

### 2.2. External Sensor Node

Unlike the internal node, the external node is battery-powered and needs to save energy as much as possible to increase battery life. The target lifetime was fixed to at least 3000 h that corresponds with the required calibration interval for electrochemical sensors. For this reason, the data are acquired and transmitted from sensors every 1 h, an interval that is acceptable considering that this node measures the environmental air quality outside the building of the plant. The electrochemical sensor’s front end has a very low energy demand and it is maintained continuously operating since electrochemical sensors require a long power-up time, before reaching the nominal measurement accuracy.

The MCU is programmed exploiting low-power programming techniques, providing a sleep routine to periodically activate the MCU only for short time intervals functional to the accomplishment of the firmware instructions, with a run mode of approximately 120 s every hour.

When the MCU is in run mode it samples the sensors, processes the data, and transmits them via LoRaWAN the samples collected, fulfilling the monitoring task of 24 readings per day. In the following Table 3, the power consumption analysis of the external node is reported.

Additionally, the external module for the measurement of particulate matters, connected by a serial link to the microcontroller, is turned on every hour for 1 min to obtain a sufficiently accurate measurement and then is powered off.

### 2.3. Gas Analyzer

**Figure 3:**
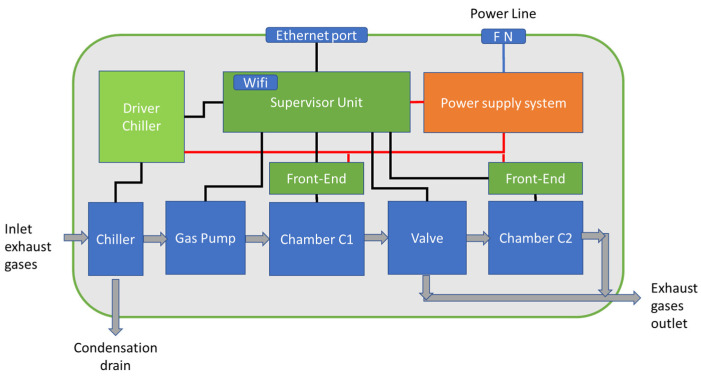
A block diagram of the gas analyzer.

The gas analyzer structure is shown in Figure 3 and its design is based on the idea presented in [20], which exploits the series of two measurement chambers (C1 and C2 in Figure 3) containing the gas sensors connected through a safety valve (valve in Figure 3). The second chamber, C2, contains the gas sensors actually used for monitoring purposes, which are the same electrochemical sensors used in the sensor nodes, whereas chamber C1 is used as a protection device. In fact, sensors in C1 are used to detect if the target gas concentrations overcome specified thresholds and, in those cases, the valve is actuated and deviates the gas flow from the measurement chamber C2. In [20], the chamber C1 contained a chemoresistive sensor, used to protect an electrochemical sensor in C2. The chemoresistive sensor was used to detect concentrations of CO larger than the electrochemical sensor on a full scale. In the design presented in this work, only electrochemical sensors are used: a large-range electrochemical sensor in the first chamber and a low-range, more accurate sensor in the second chamber. Using electrochemical sensors in chamber C1, in place of chemoresistive ones, improves performances in terms of gas selectivity. The developed two-chamber structure solves the previously introduced issue, i.e., when exposing electrochemical sensors to gas concentrations pulses largely exceeding their full scale very long recovery times are needed. Since in combustion processes, like the one considered in this application, the only target compound that is expected to experience large variations of the concentration values during operations is CO, this strategy is used only for CO monitoring (two sensors, an Alphasense CO-AX named hereafter CO_Hi and an Alphasense CO-A4 named CO_Lo are placed in C1 and C2, respectively).

**Figure 4:**
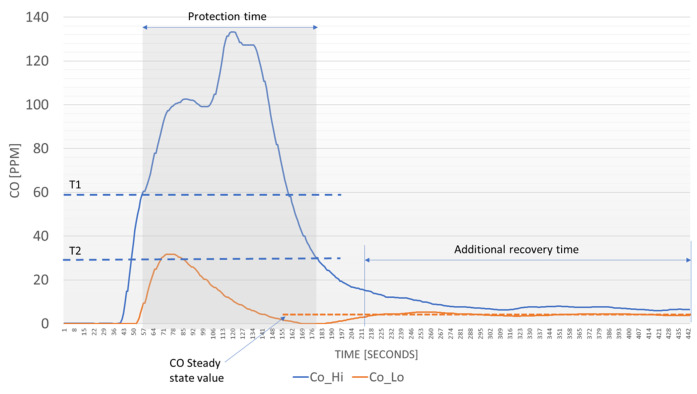
Carbon monoxide concentration transient measured by the protected sensor (Co_Lo) and by the protection sensor (Co_Hi). T1 and T2 are the threshold values used to activate the proper working condition.

From the test reported in Figure 4, it is evident the benefit of the protection mechanism: the presence of a CO_Hi sensor allows for avoiding long recovery time for CO_Lo, which is the sensor used for actual monitoring. In detail, the system behavior shown in Figure 4, highlights that if the CO_Hi sensor output overcomes the threshold T1, the system deviates the gas flow from the CO_Lo sensor that can recover quickly the zero. When the CO_Hi detects a concentration lower than the threshold T2 the sensor CO_Lo is exposed again to the gas (low concentration) allowing a fast-settling time different from the CO_Hi sensor that, due to the exposition to a very high CO concentration, requires a longer recovery time. Obviously, this technique generates benefits only in the presence of fast high-concentration transients (such as the CO typical behavior in a burner) and when the concentrations of interest are below the threshold T2 set for valve intervention, which is decided according to the measurement range.

The valve system is controlled by the electronics of the analyzer as are the pump and the chiller for gas dehumidification.

**Figure 5:**
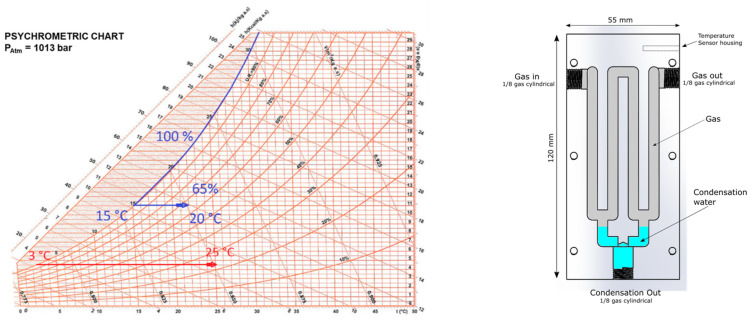
(**Left**): Psychometric chart at 1 atm showing the dehumidification process in the chiller. (**Right**): Structure of the chiller.

The gas analyzer, as shown in Figure 3, contains a gas sampling system to pump and dehumidify exhaust gases (exploiting the block called chiller) before they are introduced into the two measurement chambers. The chiller is based on an ad hoc designed thermoelectric module, inserted in a control loop that cools the gas entering the analyzer. This gas conditioning system is mandatory when working with high-temperature gases containing water, such as combustion exhausts, to avoid water condensation inside the instrument. In fact, exhaust gases at high temperatures are easily saturated by water vapor and can produce condensation when, while flowing through the measuring chambers, cool down. The thermoelectric cooler was designed and tested to cool the gases from a temperature around 100 °C and a humidity level close to 100% RH to a temperature lower than 15 °C, exploiting a temperature measurement and a feedback loop for controlling the power delivered to the Peltier cell, thus obtaining, when gases return to environmental temperature, relative humidity around 65% RH that avoid the risk of condensation inside the instrument (as shown in the psychometric chart in Figure 5). The chiller is based on a metal structure (shown in the leftmost drawing of Figure 5) that is cooled by two Peltier cells. At the bottom of this structure, a pump drains the condensed water generated by the gases.

**Figure 6:**
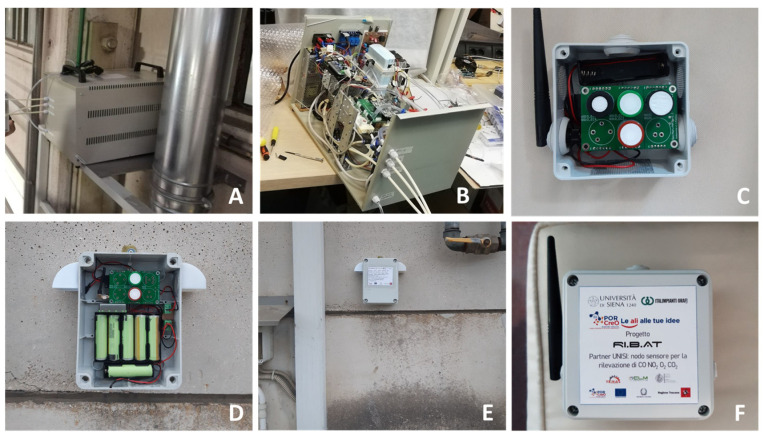
(**A**): The gas analyzer installed close to furnace exhaust gas pipeline. (**B**): A picture of the gas analyzer without the case cover. (**C**,**F**): Internal sensor node pictures. (**D**,**E**): External battery-powered sensor node pictures.

The developed devices mounted in the plant are shown in Figure 6.

## 3. Data Acquisition and Visualization

**Figure 7:**
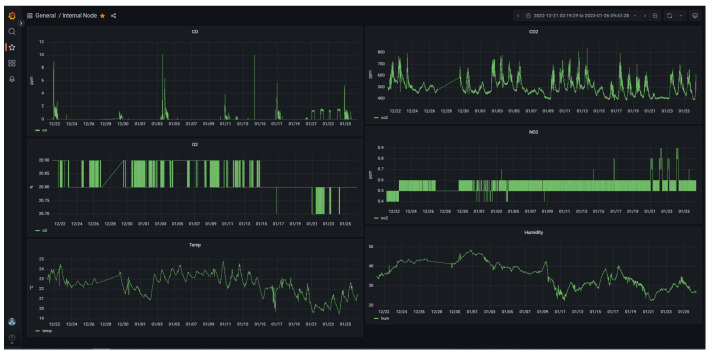
Grafana-based dashboard of the front-end showing gas concentration trends.

The data transmitted by the sensor nodes through the LoRa channel are collected by the gateway and transmitted to the remote LoRaWAN server. The data collected by the analyzer are directly sent to the server through the local network. Data are then stored in a database on the server and are accessible with a front end based on Grafana v 9.1.4, an open-source dashboard platform, as shown in Figure 7, which presents the data gathered by the internal node, CO, NO_2_, CO_2_, O_2_ concentrations, temperature, and relative humidity over a period of about 18 days.

**Figure 8:**
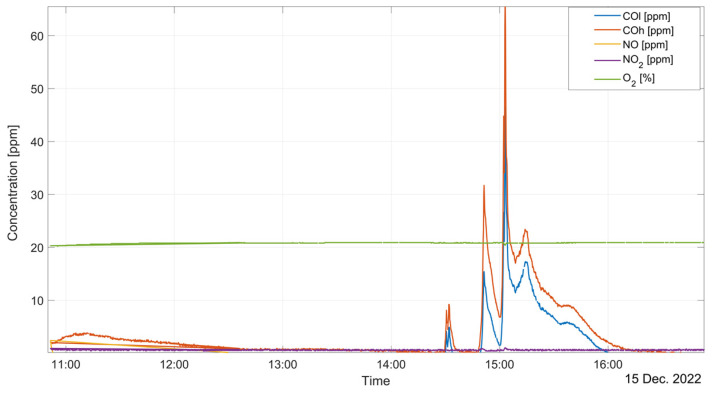
Gas concentrations measured by the gas analyzer at the melting furnace exhaust.

**Figure 9:**
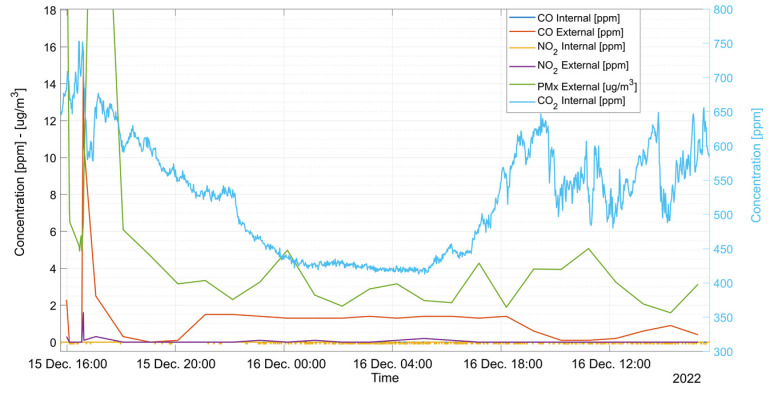
Gas concentrations measured by the external and internal nodes.

Figure 8 and Figure 9 report some examples of the outputs obtained by the complete distributed measurement system. Data are shown in two different plots: Figure 8 displays the gas concentration measured by the gas analyzer during furnace operations whereas Figure 9 shows the target gas concentrations measured by both the internal sensor node (one sample per minute) and the external node (one sample per hour) during the following day.

### Data Analysis

The data of the sensor nodes are continuously received by the server also when the furnace is not operating while the gas analyzer data are transmitted only when the furnace is working, and a melting process is in place. Sensor node data are used to monitor the air quality in the plant by transmitting the sensor readings with a pre-set duty cycle; moreover, they send immediate data to the server in case of alarms, i.e., when some settable thresholds are exceeded. When the furnace is operating, the data from the analyzer are also available. In this scenario, the server monitors the concentration of carbon monoxide and nitric oxides in the exhaust gases. At the same time, the oxygen concentration is also monitored to calculate the air dilution of the exhaust gases. In fact, the gases coming from the fusion processes are collected by an aspirator which dilutes them with air. This procedure has the effect of reducing the concentration of toxic compounds measured by the analyzer. As the flow rate of the aspirator varies, the concentrations of CO and NO_x_ measured in the analyzed gases also change. The measurement of oxygen, considering that the concentration of O_2_ in the air is fixed at 20.8% in volume and that in the fumes of fusion, it is approximately 0%, makes it possible to calculate the dilution rate. In practice, the dilution is commonly referred to as a reference value that for combustion exhausts is usually considered 15% ([O_2_]_Ref_):

(1) $$D=\frac{{20.8\%-[O_{2}]}_{Ref}}{{20.8\%-[O_{2}]}_{exhaust}}$$

[O_2_]_exhaust_ is the concentration of oxygen measured by the analyzer, using this correction the concentration of CO given the measured concentration by the analyzer [CO]_exhaust_ can be derived as (the same is applied for NOx):

(2) $$\left[CO\right]={D\left[CO\right]}_{exhaust},\;\;\;\left[NO\right]={D\left[NO\right]}_{exhaust},\;\;\;[{NO}_{2}]={D[{NO}_{2}]}_{exhaust}$$

**Figure 10:**
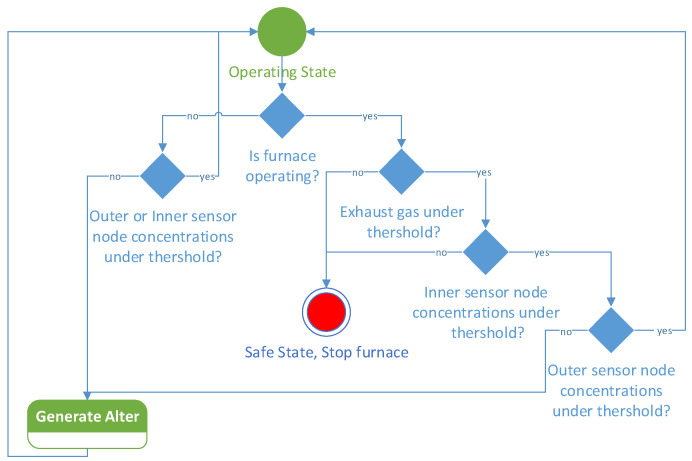
UML state machine diagram of the safe decision algorithm implemented on the server.

Once these pre-processing calculations have been applied, the gas analyzer data can be used for two main purposes. One is to monitor that fusion processes are running correctly and do not deviate from the expected behavior. The second is to promptly detect dangerous high emissions of toxic compounds whose leakage can poison the air inside the plant and expose worker to risk. In Figure 10, the state machine implementing the decision algorithm is illustrated. The operating state is the normal operation of the plant when no anomalies are detected. In case of anomalies generated by the melting furnace, detected by the analyzer of the exhaust gases or by the internal sensor node, a safe state is triggered, and the furnaces can be stopped.

In all other cases of anomalies detected by sensors, an alarm is generated on the server. This alarm can be notified to operators or to other emergency systems present in the plant.

## 4. Conclusions and Future Developments

In this work, a system to monitor the air quality in a plant where a melting furnace is operating is presented. The system is fully custom designed and meets two basic requirements: allowing the use of low-cost commercial sensors for harsh environment applications and guaranteeing the integration of the system with existing data management and supervision systems. The proposed architecture is not based on software and hardware proprietary solutions allowing a high versatility and portability to different scenarios. The most challenging aspects concerning the design of the system were essentially two. The first one concerns the design of both hardware and firmware of the battery-powered sensor node to grant low-power consumption, thus ensuring a battery lifetime of at least 3000 h. This performance was reached with a low-power design of the electronics but also by choosing the sampling and transmitting intervals. The other problem was the design of the gas analyzer using commercial electrochemical sensors. The problem was solved with the presented sensor protection technique.

The designed system is flexible and can be applied to other use cases where a burner or other source of exhaust gases inside a plant is present. The sensors can be easily installed in different points of the plant thanks to the long-range wireless connectivity and the possibility to exploit battery operating power. The modular structure of the gas analyzer allows for the choice of different sensors depending on the substances of interest.

Regarding the costs and the technology readiness level (TRL) reached by the designed system, the sensor nodes reached a good level of engineering. The electronic design, even if required a strong effort, in particular, for the reduction of power consumption, fully meets the needs (TRL 6–7). The cost of the device obviously depends on the quantity produced and on other aspects of industrialization, at the moment the cost of the components is around EUR 250 per sensor node.

The gas analyzer design was harder due to the presence of lots of different devices, electromechanical parts, and many heterogeneous components that required a more sophisticated integration phase. From the performance point of view, the instrument satisfies the requirements, but it needs a longer engineering phase to optimize the costs and improve the reliability to be eventually ready for the market (TRL 4–5). The cost of the components required for this instrument is at the moment around EUR 2000.

Other future developments of the systems will also concern the server side. The availability of data both from the furnace and from the air quality sensors, will allow the use of machine learning-based algorithms to detect the insurgence of anomalies and act in preventive maintenance operations. Moreover, an installation of a larger number of environmental sensors will increase the quality of the data collected by the system. In particular, the introduction of at least two sensors for each type (external and internal), will increase the reliability of the measurements and will be used as a source of alarm in case of calibration loss of fault of one of the nodes.

## Figures and Tables

**Table 1:** Comparison of different gas compound measurement techniques.

Type	Cost	Typical Accuracy	Selectivity	Calibration Retention	Power Consumption	Availability on the Market
Chemoresistive (Toxic)	Low	Low	Poor	Good	High	Good
Electrochemical (Toxic and Oxygen)	Medium	Good	Good	Good	Low	Good
NDIR (Carbon dioxide)	Medium	High	High	Poor	High	Good
UV (Toxic, Carbon dioxide)	High	High	High	Poor	High	Poor
Laser Scattering (Particulate Matters)	Low	Good	Good	Good	High	Good

**Table 2:** List of sensors used in the developed system.

Target Compound	Sensor Model	Range	Resolution/Sensitivity	Type	Device
CO ^1^	Alphasense CO-A4	0–500 ppm	200–400 nA/ppm	Electrochemical	Sensor Node
CO	Alphasense CO-AX	0–2000 ppm	55–100 nA/ppm	Electrochemical	Gas Analyzer
NO	Alphasense NO-A1	0–250 ppm	320–480 nA/ppm	Electrochemical	Gas Analyzer
NO_2_	Alphasense NO2-A43F	0–20 ppm	175–500 nA/ppm	Electrochemical	Sensor Node/Gas Analyzer
O_2_	Alphasense O2-A1	0–25%	9–11.5 mA/%	Electrochemical	Sensor Node/Gas Analyzer
CO_2_	Alphasense IRC-A1	0–5000 ppm	20 ppm (resolution)	NDIR	Sensor Node
Particulate matters (PM10)	Sensirion SPS30	0–1000 mg/m^3^	25 mg/m^3^ (resolution)	Optical (digital)	Sensor Node

^1^ Four electrode sensors for low concentrations (<50 ppm).

**Table 3:** Power consumption analysis of external battery-powered node: low dropout linear (LDO) regulator used to derive the 5 V from battery voltage).

Parameter	Value
Transmitting and measurement period	3600 s
Active time	30 s
Current consumption when active	70 mA
Standby current consumption	1.2 mA
Working voltage, derived from battery by an LDO	5 V
Battery voltage	8 V
Battery capacity	70 Wh
LDO quiescent current	0.06 mA
Average power consumption	8.87 mW
LDO average quiescent power consumption	0.18 mW
LDO average power dissipation due to load current	5.32 mW
Average total power consumption	14.37 mW
Estimated battery lifetime	4872 h

## Data Availability

Not applicable.

## Abbreviation

C1	Measurement Chamber 1 of the gas analyzer
C2	Measurement Chamber 2 of the gas analyzer
CEM	Continuous emission monitoring systems
CO_Hi	CO sensor for High concentrations
CO_Lo	CO sensor for Low concentrations
DC	Direct current
LDO	Low dropout linear voltage regulator
LoRa	Long range technology
MCU	Micro controller unit
NDIR	Non-dispersive infrared
PM10	Particulate matter with diameter smaller than 10 μm.
RH	Relative humidity
UV	Ultraviolet
TRL	Technology readiness level
VOCS	Volatile organic compounds

## References

[1] Tsujita W., Yoshino A., Ishida H., Moriizumi T. (2005). Gas sensor network for air-pollution monitoring. Sens. Actuators B Chem..

[2] Diamond D., Coyle S., Scampagnani S., Hayes J. (2008). Wireless sensor networks and chemo-/biosensing. Chem. Rev..

[3] Arshak K., Moore E., Lyons G.M., Harris J., Clifford S. (2004). A review of gas sensors employed in electronic nose applications. Sens. Rev..

[4] Dhall S., Mehta B.R., Tyagi A.K., Sood K. (2021). A review on environmental gas sensors: Materials and technologies. Sens. Int..

[5] Saini J., Dutta M., Marques G. (2020). A comprehensive review on indoor air quality monitoring systems for enhanced public health. Sustain. Environ. Res..

[6] Mead M.I., Popoola O.A.M., Stewart G.B., Landshoff P., Calleja M., Hayes M., Baldovi J.J., McLeod M.W., Hodgson T.F., Dicks J. (2013). The use of electrochemical sensors for monitoring urban air quality in low-cost, high-density networks. Atmos. Environ..

[7] Li Q., Zeng W., Li Y. (2022). Metal oxide gas sensors for detecting NO_2_ in industrial exhaust gas: Recent developments. Sens. Actuators B Chem..

[8] Sheik S., Marco S., Huerta R., Fonollosa J. (2014). Continuous Prediction in Chemoresistive Gas Sensors Using Reservoir Computing. Procedia Eng..

[9] Martin C.R., Zeng N., Karion A., Dickerson R.R., Ren X., Turpie B.N., Weber K.J. (2017). Evaluation and environmental correction of ambient CO_2_ measurements from a low-cost NDIR sensor. Atmos. Meas. Tech..

[10] Kingsy Grace R., Manju S. (2019). A Comprehensive Review of Wireless Sensor Networks Based Air Pollution Monitoring Systems. Wirel. Pers. Commun..

[11] González E., Casanova-Chafer J., Romero A., Vilanova X., Mitrovics J., Llobet E. (2020). LoRa Sensor Network Development for Air Quality Monitoring or Detecting Gas Leakage Events. Sensors.

[12] Marinov M.B., Topalov I., Gieva E., Nikolov G. Air quality monitoring in urban environments. Proceedings of the 2016 39th International Spring Seminar on Electronics Technology (ISSE).

[13] Liu J., Chen Y., Lin T., Chen C., Chen P., Wen T., Sun C., Juang J., Jiang J. (2012). An Air Quality Monitoring System for Urban Areas Based on the Technology of Wireless Sensor Networks. Int. J. Smart Sens. Intell. Syst..

[14] Spirjakin D., Baranov A., Karelin A., Somov A. Wireless multi-sensor gas platform for environmental monitoring. Proceedings of the 2015 IEEE Workshop on Environmental, Energy, and Structural Monitoring Systems (EESMS) Proceedings.

[15] Arroyo P., Herrero J.L., Suárez J.I., Lozano J. (2019). Wireless Sensor Network Combined with Cloud Computing for Air Quality Monitoring. Sensors.

[16] Kwon J., Ahn G., Kim G., Kim J.C., Kim H. A study on NDIR-based CO_2_ sensor to apply remote air quality monitoring system. Proceedings of the 2009 ICCAS-SICE.

[17] Cappelli I., Fort A., Pozzebon A., Tani M., Trivellin N., Vignoli V., Bruzzi M. (2022). Autonomous IoT Monitoring Matching Spectral Artificial Light Manipulation for Horticulture. Sensors.

[18] Yasuda T., Yonemura S., Tani A. (2012). Comparison of the Characteristics of Small Commercial NDIR CO_2_ Sensor Models and Development of a Portable CO_2_ Measurement Device. Sensors.

[19] Garcia-Romeo D., Fuentes H., Medrano N., Calvo B., Martínez P.A., Azcona C. A NDIR-based CO_2_ monitor system for wireless sensor networks. Proceedings of the 2012 IEEE 3rd Latin American Symposium on Circuits and Systems (LASCAS).

[20] Addabbo T., Bardi F., Cioncolini S., Fort A., Mugnaini M., Parri L., Vignoli V., De Gloria A. (2019). Multi-sensors Exhaust Gas Emission Monitoring System for Industrial Applications. Applications in Electronics Pervading Industry, Environment and Society, Proceedings of the ApplePies 2017.

